# Androgen-Mediated T-cell Suppression Drives Sex Bias in Head and Neck Squamous Cell Carcinoma

**DOI:** 10.1158/2767-9764.CRC-26-0244

**Published:** 2026-11-03

**Authors:** Quinn Hopen, Samantha Antol, Carly J. Amato, Benjamin C. Duncan, Jonathan T. Busada, Lei Wang, Gangqing Hu, Jennifer Franko

**Affiliations:** 1 Department of Microbiology, Immunology, and Cell Biology, West Virginia University School of Medicine, Morgantown, West Virginia.; 2 Department of Dermatology, West Virginia University School of Medicine, Morgantown, West Virginia.; 3 WVU Cancer Institute, West Virginia University, Morgantown, West Virginia.; 4 Department of Research, West Virginia University School of Dentistry, Morgantown, West Virginia.

## Abstract

Head and neck squamous cell carcinoma (HNSCC) exhibits a strong sex bias. However, the mechanisms underlying this disparity remain unclear. Using the four core genotypes (FCG) mouse model to uncouple gonadal sex from sex chromosome complement influences, we investigated the contributions of sex hormones and sex chromosomes to HNSCC development and antitumor immunity. Male-biased tumor growth was confirmed in 4-nitroquinoline-1-oxide (4-NQO)–induced oral lesions and orthotopic MOC1 tumors in both wild-type (WT) and FCG mice. Gonadal sex, rather than sex chromosomes, was identified as a primary driver of male tumor growth. However, the interactive effects of gonadal sex and possession of an XX chromosome complement limited MOC1 tumor growth in FCG females. Mechanistically, *in vitro* dihydrotestosterone (DHT) exposure did not increase MOC1 proliferation and induced only modest transcriptomic changes, suggesting that tumor-extrinsic androgen receptor (AR) signaling promotes male tumor growth *in vivo*. Notably, sex differences in MOC1 tumor growth were eliminated in T-cell receptor α–knockout (TCRαKO) mice, implicating sex-specific T-cell regulation in HNSCC sex biases. Interestingly, castration reduced MOC1 tumor burden and enhanced both T-cell activation and infiltration into male MOC1 tumors, whereas greater CD8^+^ T-cell responses in XX-possessing FCG females were suggestive of the interactive influence of sex chromosomes and sex hormones on female antitumor immunity. Together, these results identify sex-specific T-cell regulation as a critical factor in HNSCC risk and highlight androgens as a potential therapeutic target to enhance tumor-specific immunity and immunotherapeutic efficacy in patients with HNSCC.

**Significance:**

Sex-specific antitumor T-cell immunity is identified as a key promoter of HNSCC sex biases, providing a promising therapeutic target to enhance the immunotherapeutic efficacy in patients with HNSCC.

## Introduction

Head and neck squamous cell carcinoma (HNSCC) is the seventh most common cancer worldwide with approximately 890,000 new diagnoses annually (1). It exhibits marked sex differences in incidence and survival. In 2024, the age-adjusted incidence rate was 3 times higher in males versus females. Although gender-specific lifestyle risks (e.g., smokeless tobacco use) exist (2), such factors likely only add to the influence of other biological factors, including sex hormones and sex chromosome complement, to modulate HNSCC risk.

Historically, tumor-intrinsic sex hormone signaling has been the primary focus of research investigating the underlying cause of cancer sex biases. It is now well established that sex hormones can directly modulate the growth and invasiveness of reproductive organ tumors, including breast and prostate cancers, as well as certain nonreproductive organ cancers, such as bladder and liver cancers. The role of sex hormones in solid tumors is complex as it varies across tumor types, sex hormone receptor expression levels, and signaling. In general, tumor-intrinsic androgen receptor (AR) signaling promotes tumor cell proliferation and survival, although some reports have demonstrated its metastasis-suppressing effects (3). Estrogen-mediated effects are receptor subtype dependent, with estrogen receptor (ER) α and ERβ signaling acting as opposing forces in estrogen-responsive cancers. Whereas ERα promotes tumor cell proliferation and survival, ERβ inhibits proliferation and promotes apoptosis. Variations in ERα/ERβ expression levels are often predictive of patient prognoses (4). In addition to sex hormones, a cell’s sex chromosome complement (XX vs. XY) has also been associated with cancer risk. Although mutations in X-linked tumor-suppressor genes or oncogenes increase risk in males due to hemizygosity, the biallelic expression of “escape from X-inactivation tumor-suppressor” genes has been implicated in conferring protection in females (5, 6).

Given the critical role the tumor immune microenvironment (TIME) plays in regulating tumor onset and progression, there is growing interest in determining whether the sex-specific regulation of antitumor immunity also influences cancer risk. Immune sex biases are well characterized in response to infection, vaccination, and autoimmune disease and again also reflect the influence of both sex hormones and sex chromosome complement (7). Numerous studies have illustrated the ability of estrogens to affect T-cell immunity. ERα signaling is known to promote regulatory T-cell (Treg) infiltration into tumors and is correlated with reduced antitumor CD4^+^ and CD8^+^ T-cell infiltration (8, 9). In contrast, ERβ signaling is reported to both enhance CD8^+^ T-cell activity through a phosphotyrosine switch and inhibit CD8^+^ T-cell activity by upregulating PD-L1 expression (10, 11). Multiple recent reports have highlighted the suppressive impact of AR signaling on T-cell immunosurveillance via its ability to (i) potentiate the terminal exhaustion of T cells, (ii) suppress T-cell effector functions, and (iii) downregulate the dendritic cell licensing function of CD4^+^ T helper cells (12–14).

Given that many immune sex biases exist prior to puberty and after menopause onset, sex chromosome complement has become an increasingly appreciated sex-specific modulator of immune activation and differentiation (15, 16). In comparison with other chromosomes, the X chromosome encodes for the largest number of immune-related genes in both the human and mouse genomes (17). In most somatic cells, X-linked gene expression is typically balanced between males and females via the process of X chromosome inactivation (XCI). However, XCI is differentially regulated in lymphocytes and multiple X-linked immune-related (XLIR) genes have now been demonstrated to escape XCI (18). The resulting biallelic expression and consequent XLIR gene dosage effects have been reported to enhance lymphocyte activation in XX-possessing cells in several recent studies (19, 20), including our own (21). Although recent studies have begun to focus on androgen-mediated impacts on the TIME, less is known about the role sex chromosome complement plays in regulating HNSCC antitumor immunity and if sex hormones and sex chromosomes work in collaboration to promote sex biases.

The ability to investigate the role of sex chromosome complement in sex-biased phenotypes has become feasible because of the development of the novel four core genotypes (FCG) mouse model, which consists of XX and XY gonadal females with ovaries and XX and XY gonadal males with testes (22, 23). The model was developed by deleting the testis-determining factor, *Sry*, from the Y chromosome and placing it as a transgene on autosome 3. The uncoupling of gonadal sex from sex chromosome complement allows for the identification of phenotypes influenced by XX or XY sex chromosome complement, gonadal secretions, and potential interactions between the two factors. FCG mice have been utilized to identify sex chromosome–dependent influences in a variety of sexually dimorphic phenotypes, including brain development, behavior, adiposity, and metabolism, as well as in sex-biased immunity, including autoimmunity, infection, and vaccine responses (24–26).

In the current study, we evaluated the underlying mechanisms of HNSCC sex biases in wild-type C57BL/6J and FCG mice. Generation of orthotopic MOC1 syngeneic tumors in FCG mice specifically allowed for the identification of sex chromosome–dependent phenotypes that were dependent on an immune cell’s sex chromosome complement. The influence of gonadal secretions was evaluated using the FCG model, gonadectomy (GDX) surgeries, and *in vitro* exposure studies. In males, MOC1 tumor growth was found to be driven primarily by gonadal sex rather than sex chromosome complement. Females, however, benefited from the interactive effects of gonadal sex and the sex chromosome complement (XX > XY) of their immune cells. Similar results were achieved using the 4-nitroquinoline-1-oxide (4-NQO) exposure model. Although MOC1 cells expressed AR, *in vitro* dihydrotestosterone (DHT) exposure did not increase MOC1 proliferation and induced only modest transcriptomic changes, suggesting that androgen-mediated effects on tumor growth are likely tumor extrinsic. Notably, sex differences in MOC1 tumor growth were eliminated in T cell–deficient mice, implicating sex-specific T-cell regulation in HNSCC sex biases. Castration reduced MOC1 tumor growth and enhanced both T-cell activation and infiltration into male MOC1 tumors. Ovariectomy did not alter MOC1 tumor growth or antitumor immunity in female C57BL/6J mice. However, FCG females possessing an XX sex chromosome complement benefited from the interactive influence of gonadal sex and sex chromosomes, demonstrating reduced tumor growth and enhanced antitumor immunity. Together, these results identify sex-specific T-cell regulation as a critical factor in HNSCC risk and highlight androgens as a potential therapeutic target to enhance tumor-specific immunity and immunotherapeutic efficacy in patients with HNSCC.

## Materials and Methods

### Cell culture

The mouse oral squamous cell carcinoma (MOC1; RRID:CVCL_ZD32) cell line (Kerafast, cat. #EWL001-FP) was maintained per the manufacturer’s recommendation in a 2:1 mixture of Iscove’s modified Dulbecco’s medium and Ham’s F-12 (HyClone, cat. #sh30228.02 and #sh30026.01) supplemented with 5% heat-inactivated fetal bovine serum (FBS), 100 U/mL penicillin–streptomycin, 5 ng/mL human recombinant epidermal growth factor (Millipore, cat. #01-107), 400 ng/mL hydrocortisone (Sigma-Aldrich, cat. #H0135), and 5 μg/mL insulin (Sigma-Aldrich, cat. #I6634; ref. 27). For *in vitro* DHT (100 nmol/L; Steraloids, cat. #A2570-000) exposure studies, media was supplemented with charcoal-stripped FBS (Corning, cat. #35-072-CV). Cells were thawed (liquid nitrogen), passed at least twice before use, and were not used beyond passage 12. No further authentication or pathogen testing was performed on MOC1 cells following their acquisition from Kerafast (2022).

### Animal studies

C57BL/6J (RRID:IMSR_JAX:000664), T-cell receptor α knockout (TCRαKO; B6.129S2-*Tcra*^*tm1Mom*^/J; RRID:IMSR_JAX:002116), and XY^−^*Sry* [B6.Cg-Tg(Sry)2Ei Sry^dl1Rlb^/ArnoJ; RRID:IMSR_JAX:010905 mice were purchased from The Jackson Laboratory. FCG mice were generated by breeding XY^−^*Sry* males with C57BL/6J females, producing XX and XY gonadal males (XXM and XYM, respectively) and females (XXF and XYF, respectively; Fig. 2A; refs. 22, 23). Genotyping was performed by PCR amplification of *Myo*, *Sry*, and *Ymt* (Supplementary Table S1). Mice were housed under specific pathogen–free conditions (12-hour light–dark cycle, *ad libitum* food and water). Purchased mice were acclimated for ≥1 week prior to use. All procedures were in compliance with federal/institutional guidelines and approved by the West Virginia University (WVU) Institutional Animal Care and Use Committee (protocol #2108045855). For chemical carcinogenesis, 4-NQO (Sigma-Aldrich, cat. #N8141) was dissolved in DMSO and administered in drinking water (100 μg/mL) for 16 weeks, as previously described (28, 29). Following 16 weeks of 4-NQO exposure, mice were switched to normal drinking water for an additional 7 weeks. Beginning at 14 weeks after initial 4-NQO exposure, animals were monitored weekly for weight loss and the number and size of 4-NQO–induced lesions. Tumor volumes [(L × W × H)/2] and tumor areas (L × W) were measured using calipers and calculated.

For syngeneic tumors, MOC1 cells (6 × 10^4^ cells in 30 μL sterile saline) were orthotopically injected into the lateral tongue (30). Briefly, mice were anesthetized with isoflurane, and tongues were gently pulled out with forceps. Using a 1-mL insulin syringe and attached 30-gauge needle, MOC1 cells were injected 1.5 mm from the tip of the tongue so that a bulbous mass formed at the center of the tongue. Beginning on day 7 after injection, body weight and oral tumor evaluations were performed weekly. Tumor volumes [(L × W × H)/2] and tumor areas (L × W) were measured using calipers and calculated.

GDX surgeries were performed as described in (21). Briefly, mice were anesthetized with isoflurane. Castrations were performed by making an incision through the skin along the midline of the scrotal sac and then through the membrane of each testis. Ovariectomies were performed by making an incision through the skin and underlying abdominal wall. The sex organs were isolated and removed with heated forceps to cauterize the vas deferens and blood vessel or transect the tip of the uterine horn and cauterize the blood vessels. The abdominal wall was closed with a suture, and skin incisions were closed with a wound clip. Sham operations followed the same procedure, but sex organs were left intact. GDX or sham-operated (sham) animals were housed for 2 to 4 weeks before experimental use.

### Immunohistochemistry

Formalin-fixed, paraffin-embedded MOC1 tumor sections (5 μm) were deparaffinized with CitriSolv (DeconLabs, cat. #1601) and underwent antigen retrieval by heating sections in Immuno Retriever Citrate Buffer (Bio SB, cat. #BSB-0023) in the TintoRetriever Digital Pressure Cooker (Bio SB, cat. #BSB-7087). Sections were blocked in 4.5% normal goat serum at 25°C for 30 minutes. Sections were incubated overnight at 4°C with anti-AR or isotype control antibodies (Table 1) using the VEXTASTATIN ABC-HRP Kit (Vector Labs, cat. #PK-6101). Detection was performed utilizing the ImmPACT 3,3′-diaminobenzidine (DAB) Substrate Kit (Vector Labs, cat. #SK-4105) according to the manufacturer’s recommendations, and sections underwent hematoxylin counterstaining for 5 minutes. Finally, tissues were dehydrated, and glass coverslips were mounted using distyrene, plasticizer, and xylene mounting medium (Electron Microscopy Sciences, cat. #13510). Images were acquired using an Olympus VS120 Slide Scanner (RRID:SCR_018411). Percent positive area was determined using QuPath (v0.6.0; RRID:SCR_018257) to separate stains (DAB: 0.28, 0.556, and 0.783; hematoxylin: 0.705, 0.629, and 0.329; and residual: 0.521, −0.773, and 0.363).

**Table 1.** Flow cytometry and imaging antibodies and dyes.

Marker	Clone	Fluorophore	Manufacturer	Cat. #	RRID
LIVE/DEAD Yellow	—	—	Invitrogen	L34959	
CD45	30-F11	APC-Cy7	BioLegend	103116	RRID:AB_312981
CD3	17A2	PerCP-Cy5.5	BioLegend	100218	RRID:AB_1595492
CD4	GK1.5	FITC	BD Biosciences	553729	RRID:AB_395013
CD8a	53–6.7	PE	BD Biosciences	553033	RRID:AB_394571
PD-1	29F.1A12	PE/Fire 810	BioLegend	135253	RRID:AB_2910293
LAG-3	C9B7W	BV785	BioLegend	125219	RRID:AB_2566571
Granzyme B	QA16A02	APC	BioLegend	372204	RRID:AB_2687028
IFN-γ	XMG1.2	BV711	BioLegend	505836	RRID:AB_2650928
CD274 (PD-L1)	10F.9G2	PE	BioLegend	124308	RRID:AB_2073556
H-2Kb	AF6-88.5	FITC	BioLegend	116505	RRID:AB_313732
AR	EPR1535(2)	—	Abcam	ab133273	RRID:AB_11156085
Rabbit IgG isotype control	EPR25A	—	Abcam	ab172730	RRID:AB_2687931

Abbreviations: APC, allophycocyanin; Cat #, catalog number.

### Immunocytochemistry

MOC1 cells (1 × 10^5^) were seeded in 12-well plates containing sterile coverslips overnight and then treated with 100 nmol/L DHT or vehicle control (absolute ethanol) for 3 hours. Cells were fixed with 10% formalin for 20 minutes, permeabilized with 0.1% Triton X-100 for 10 minutes, and blocked with 3% bovine serum albumin (BSA) for 1 hour. Cells were then incubated with anti-AR antibody at 4°C overnight (Table 1), followed by incubation with secondary antibody at 25°C for 2 hours. Nuclei were stained with Hoechst 33342 (Invitrogen, cat. #H3570) at 25°C for 5 minutes. Glass coverslips were mounted with ProLong Gold Antifade mountant (Invitrogen, cat. #P36930). Images (3–5/sample) were acquired using a Nikon A1R Confocal/N-SIM-E microscope (RRID:SCR_020317) equipped with four lasers (405, 488, 561, and 640 nm). AR nuclear translocation was analyzed using Fiji (v2.16.0; RRID:SCR_002285). Briefly, nuclear masks were generated by creating a threshold (auto threshold; default) to detect Hoechst-positive pixels. A threshold was then created to detect AR-positive pixels (65–255). AR mean fluorescence intensity was quantified and compared within the nuclear and cytoplasmic compartments [adapted from (31)].

### Cell Counting Kit 8 proliferation assay

MOC1 cells (5,000/well) were seeded in 96-well plates ± DHT. Proliferation was assessed using the Cell Counting Kit 8 (CCK-8; MedChemExpress, cat. #HY-K0301). Two hours before each indicated time point, CCK-8 reagent (10 μL/100 μL growth media) was added to each well and allowed to develop for 2 hours at 37°C. Absorbance (450 nm) was measured using SpectraMax iD3 (Molecular Devices; RRID:SCR_023920) with SoftMax Pro (v7.1; RRID:SCR_014240).

### RNA sequencing

MOC1 cells (3 × 10^5^) were seeded in six-well plates, grown to 80% confluency, and treated with DHT or control for 6 hours. RNA was isolated using TRIzol. Following quantification and quality assessment, Illumina-compatible libraries (KAPA stranded mRNA library kit; Kapa Biosystems) were prepared from 200 ng RNA. Sequencing was performed as 2 × 51 cycles on an Illumina NextSeq 2000 using a P2 flowcell (RRID:SCR_023614; Marshall Genomics Core).

RNA sequencing (RNA-seq) data analysis followed previously described procedures (32). Briefly, RNA-seq short reads were aligned to the mm10 with subread (33). Read counts against RefSeq gene annotation was summarized with FeatureCounts (34). Differentially expressed genes (DEG) were predicted by EdgeR3 [FDR < 0.05 and a fold change >1.5]. Gene set enrichment analysis (GSEA; Supplementary Table S4) was done using GSEA (v4.2.3; ref. 35) based on gene expression change, against Kyoto Encyclopedia of Genes and Genomes gene set from Molecular Signatures Database (v2023; ref. 36). Only expressed genes (Reads per kilobase million > 3 in at least one compared condition) were included in the DEG analysis and GSEA.

### Immunophenotyping

Cervical draining lymph nodes (DLN) were excised and mechanically dissociated to single-cell suspension with plastic pestles. For assessment of tumor-infiltrating leukocytes (TIL), MOC1 tumors were resected and minced using a surgical razor blade. Tumors were then enzymatically digested with 1 mg/mL collagenase D (Roche, cat. #11088858001) and 200 μg/mL DNase I (Roche, cat. #10104159001) for 30 minutes at 37°C, vortexing every 10 minutes. Tissue fragments were then dissociated through 70-μm cell strainers to generate single-cell suspensions. TILs were enriched using the EasySep Mouse CD45 Positive Selection Kit (STEMCELL Technologies, cat. #100-0350).

Fc receptors were blocked with ChromPure IgG (Jackson ImmunoResearch, cat. #015000003) for 20 minutes at 4°C, followed by surface staining with indicated antibodies or stain for 20 minutes at 4°C (Table 1). Cells were fixed with 4% paraformaldehyde (PFA) for 20 minutes at 4°C. Absolute TIL numbers were determined using AccuCount beads (Spherotech, cat. #ACBP-50-10). For intracellular protein staining, TILs were stimulated with phorbol 12-myristate 13-acetate (500 ng/mL; Sigma-Aldrich, cat. #P8139), ionomycin (1 μmol/L; Sigma-Aldrich, cat. #I3909), and GolgiStop/Plug (BD Biosciences, cat. #554724/#555029) for 4 hours at 37°C and then surface-stained as described above. Following fixation with 4% PFA, cells were permeabilized utilizing the Foxp3/Transcription Factor Staining Buffer Set (Thermo Fisher Scientific, cat. #00-5523-00) and then incubated with anti–granzyme B and anti–IFN-γ antibodies for 30 minutes at 4°C (Table 1). Flow cytometry was performed on BD LSRFortessa (RRID:SCR_018655) or Cytek Aurora (RRID:SCR_019826) and analyzed using FCS Express (RRID:SCR_016431).

To assess H-2Kb and PD-L1 surface expression on MOC1 cells, cells were seeded and allowed to attach overnight. Next, cells were treated with DHT or vehicle ± IFN-γ (25 ng/mL; BioLegend, cat. #575304) for 24 hours. Cells were harvested with Accutase (Innovative Cell Technologies, cat. #AT104), and surface marker staining was performed as described above. Flow cytometry was performed on BD LSRFortessa or Cytek Aurora and analyzed using FCS Express.

### Statistical analysis

Data were analyzed in GraphPad Prism (v10.6.0; RRID:SCR_002798) and presented as mean ± SEM. Statistical significance was defined as *P* < 0.05. Each data point represents one mouse unless otherwise indicated. Statistical tests included an unpaired *t* test or one-way/two-way ANOVA with appropriate *post hoc* testing, as specified in figure legends. Survival analysis was performed using the log-rank test. In FCG studies, two-way ANOVA was used to assess main and interactive effects of sex chromosome complement and gonadal sex (23, 24). Main-effect graphs were generated by plotting the predicted least squares means of XX versus XY and gonadal male versus female based on two-way ANOVA analyses.

## Results

### Gonadal sex is the predominant sex determinant promoting male biases in preclinical HNSCC models

**Figure 1.**
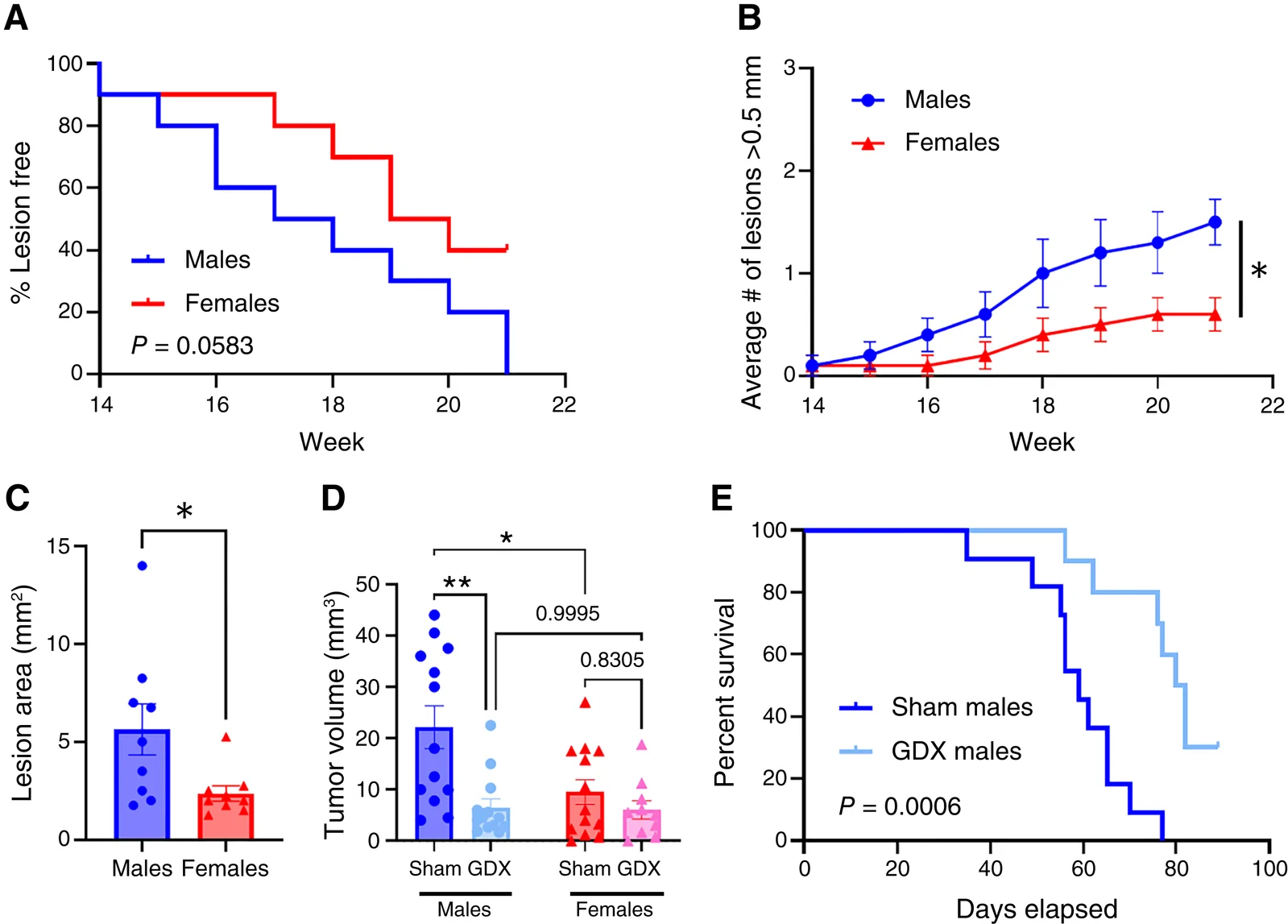
Confirmation of sex biases in preclinical HNSCC models. **A,** Disease-free survival in male and female C57BL/6J mice following 4-NQO exposure, measured as the time to first detectable lesion, and (**B**) the number of 4-NQO–induced lesions per mouse were assessed weekly. **C,** 4-NQO–induced lesion area measured at 22 weeks. **D,** Male and female MOC1 tumor volumes in sham-operated and GDX mice, 6 weeks after tumor induction. **E,** Overall survival of MOC1 tumor-bearing sham and GDX males. **A**, **B**, and **E,***n* ≥ 9 mice per group. For **C** and **D**, data are presented as the mean ± SEM with each data point representing an individual mouse. Data are representative of ≥2 independent experiments. Statistical analyses by the (**A** and **E**) log-rank test, (**B** and **C**) unpaired *t* test, or (**D**) two-way ANOVA followed by the Tukey multiple comparisons test. *, *P* < 0.05; **, *P* < 0.01.

To investigate the biological basis of HNSCC sex biases, tumorigenesis was evaluated in male and female C57BL/6J mice using two preclinical models: 4-NQO chemical carcinogen–induced oral lesions and orthotopic MOC1 syngeneic tumors. In the 4-NQO model, which induces oral lesions that reproduce the stepwise histologic progression of human patients, males demonstrated a trend toward reduced disease-free survival compared with females (Fig. 1A; *P* = 0.0583). Whereas all males developed measurable premalignant lesions (≥0.5 mm diameter), only 60% of females did so by the experimental endpoint (Supplementary Table S2). Of the affected mice, males developed more lesions (Fig. 1B), with a mean area approximately 2.4 times greater than that observed in females (Fig. 1C). Consistent with these findings, males also developed larger tumors in the orthotopic MOC1 syngeneic tumor model (Fig. 1D). To evaluate the role of sex hormones in MOC1 tumor growth, GDX surgeries were performed prior to MOC1 tumor induction. At 6 weeks, a time point chosen to allow for the greatest amount of tumor growth without reaching humane endpoint criteria in male tumor-bearing mice, no difference in MOC1 tumor size was noted in sham-operated versus ovariectomized females (Fig. 1D). In contrast, castration resulted in more than a threefold reduction in MOC1 tumor volumes at the same time point (Fig. 1D). Castration also prolonged the overall survival of MOC1-bearing male mice (Fig. 1E), with 100% of the sham-operated males, but only 70% of GDX males, reaching humane endpoint criteria by 82 days after tumor induction, suggesting that the male-biased phenotypes were androgen dependent.

**Figure 2.**
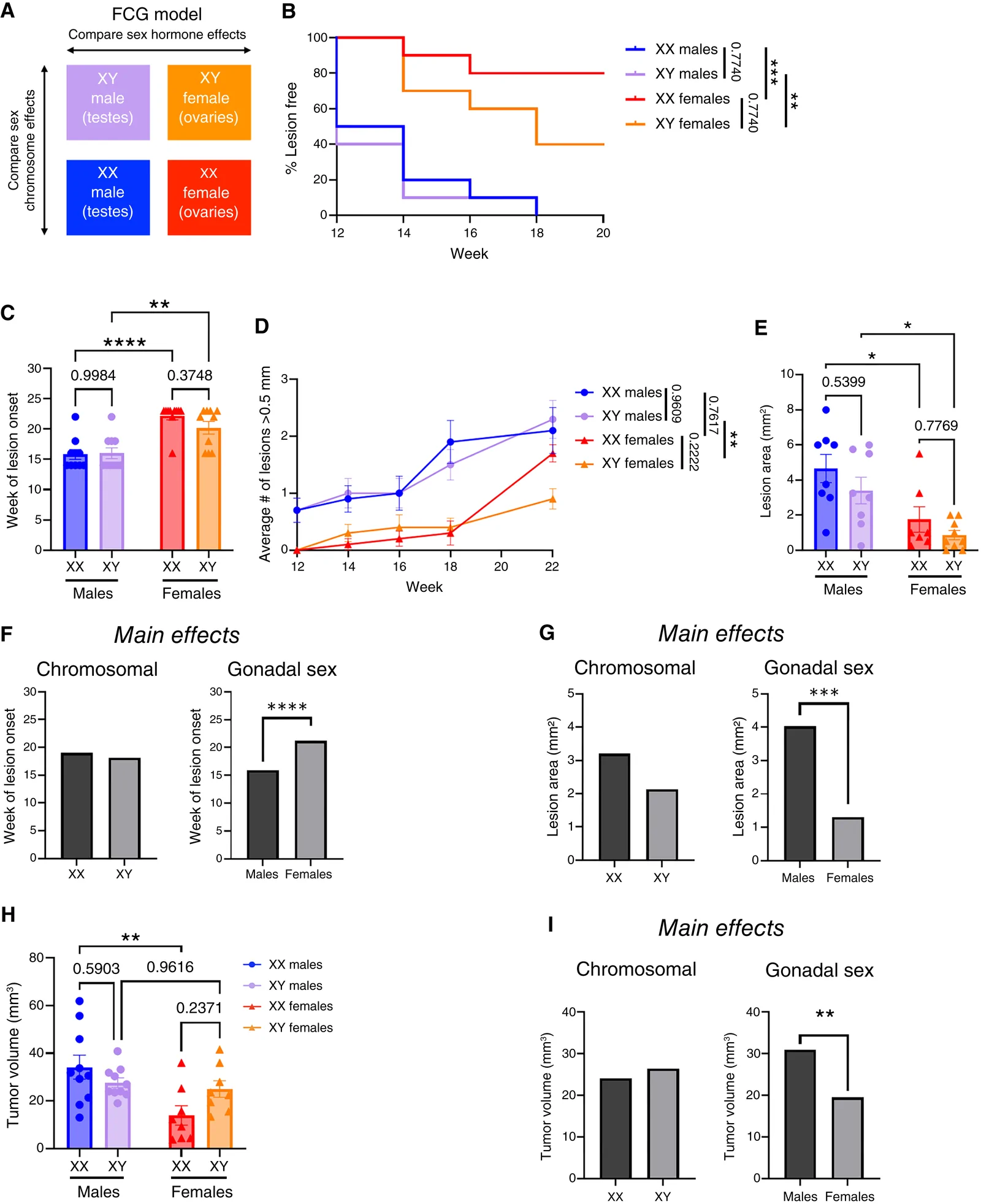
Sex hormones are the dominant sex determinant driving HNSCC sex biases. **A,** Schematic overview of the FCG mouse model, illustrating the dissociation of the sex chromosome complement from gonadal sex, with corresponding figure color coding. **B,** Lesion-free survival, (**C**) mean time to first lesion, (**D**) average number of 4-NQO–induced lesions per mouse, and (**E**) the mean lesion area of 4-NQO–induced lesions were assessed in FCG mice weekly as indicated or at 22 weeks. **H,** MOC1 tumor volumes were assessed in FCG mice 5 weeks after tumor induction. **F**, **G**, and **I,** The main effects of sex chromosome complement and gonadal sex on the variables assessed in **C**, **E**, and **H**, respectively, are presented by graphing the least squares means of each variable. **B** and **D,***n* ≥ 9 mice per group. **C**, **E**, and **H,** Data are presented as the mean ± SEM with each data point representing an individual mouse. Data are representative of ≥2 independent experiments. Statistical analyses by the (**B**) log-rank test followed by the Holm–Šidák multiple comparisons test or (**C–E** and **H**) two-way ANOVA followed by the Tukey multiple comparisons tests. *, *P* < 0.05; **, *P* < 0.01; ***, *P* < 0.001; ****, *P* < 0.0001.

To further delineate the respective contributions of sex hormones and sex chromosomes to HNSCC sex biases, we utilized the FCG model, in which gonadal sex is dissociated from chromosomal sex (Fig. 2A). Consistent with our findings in C57BL/6J mice (Fig. 1A–C), 4-NQO–exposed FCG gonadal males exhibited earlier lesion onset (Fig. 2B and C) and developed more and larger lesions (Fig. 2D and E) than gonadal females of the same chromosomal genotypes. Although males demonstrated earlier onset and larger lesions regardless of sex chromosome complement (Fig. 2B, C, and E), the number of lesions induced in response to 4-NQO exposure was greater in XYM versus XYF but not in XXM versus XXF (Fig. 2D). FCG mice fit a 2 × 2 comparison model, in which two independent main effects [sex chromosome complement (XX vs. XY) and gonadal sex (21)], as well as their interactive effect can be evaluated via two-way ANOVA (23, 24). Figure 2F and G represents the main effect of sex chromosome complement (all XX vs. all XY) and gonadal sex (all male vs. all female). Given the complex biology of the FCG model, assessment of main effects via two-way ANOVA can further aid in unmasking sex-specific interactions that contribute to observed phenotypes. Two-way ANOVA confirmed a significant main effect of gonadal sex, but not sex chromosomes, on outcomes measured in Fig. 2C and E–G and Supplementary Table S3.

In the MOC1 syngeneic tumor model, FCG XXM developed tumors approximately twice the volume of FCG XXF (Fig. 2H). No difference in tumor volume was noted between XXM versus XYM, XXF versus XYF, or XYM versus XYF (Fig. 2H). Two-way ANOVA confirmed the main effect of gonadal sex, but not sex chromosome complement, on MOC1 tumor growth (Fig. 2I; Supplementary Table S3). However, a significant interaction between gonadal sex and sex chromosome complement was observed (Supplementary Table S3), which was mainly driven by significantly larger tumors in XXM versus XXF MOC1 tumor-bearing mice. This indicates that, although male gonadal sex seems to consistently drive male-biased disease in the 4-NQO and MOC1 models, female gonadal sex and XX sex chromosome complement may act in a coordinated manner to influence MOC1 tumor growth in females.

### Tumor-intrinsic AR signaling *in vitro* has limited impact on MOC1 proliferation and transcriptional activity

**Figure 3.**
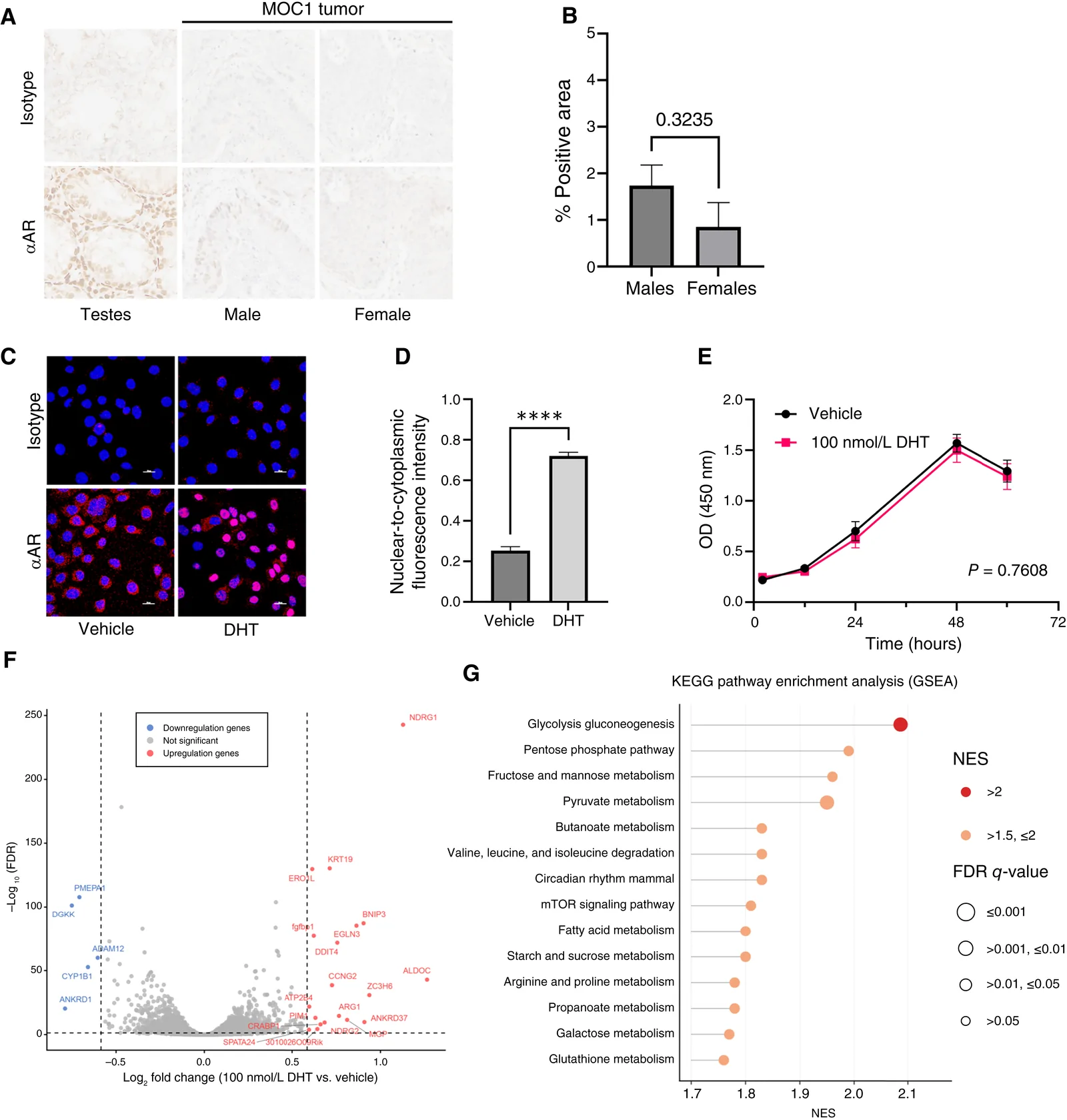
AR signaling has minimal impact on MOC1 proliferation or gene expression. **A,** AR expression was assessed by immunohistochemistry in serially sectioned MOC1 tumors (*n* = 3) collected at 8 to 10 weeks after tumor induction and (**B**) quantified. Testes served as the positive control for AR expression. **C,** Representative immunofluorescence images demonstrating AR nuclear translocation in MOC1 cells following *in vitro* DHT (100 nmol/L, 3 hours) or vehicle control exposure. **D,** Quantification is shown as the ratio of nuclear-to-cytoplasmic fluorescence intensity of AR staining in vehicle- versus DHT-treated cells. **E,** MOC1 cell proliferation in response to DHT (100 nmol/L) was assessed using the CCK-8 assay at the indicated time points. **F,** Volcano plot illustrating differential gene expression in DHT-treated (100 nmol/L, 6 hours) versus control MOC1 cells, using thresholds of log_2_ fold change > 0.585 and FDR < 0.05 (*n* = 5). The identified genes were significantly upregulated (right, red) or downregulated (left, blue) in the DHT-treated group. **G,** Bubble plot depicting gene sets enriched in DHT-exposed (100 nmol/L, 6 hours) MOC1 cells as assessed by GSEA. NES, normalized enrichment score. Data in **B**, **D**, and **E** are presented as the mean ± SEM and are representative of ≥3 independent experiments. Statistical analyses by the unpaired *t* test. ****, *P* < 0.0001. ⍺AR, anti-androgen receptor antibody; OD, optical density.

Androgens may modulate sex-specific tumor growth via tumor-intrinsic or tumor-extrinsic AR signaling pathways. The direct influence of tumor-intrinsic AR signaling on MOC1 cell proliferation and transcriptional activity was first evaluated. AR expression was confirmed in MOC1 tumors (Fig. 3A and B), and nuclear AR translocation following DHT exposure verified DHT responsiveness *in vitro* (Fig. 3C and D). Despite this responsiveness, DHT did not enhance MOC1 cell proliferation at any time point assessed (Fig. 3E), and RNA-seq identified only modest transcriptional changes following *in vitro* DHT exposure, with 24 genes being differentially expressed (19 upregulated and 5 downregulated) in DHT versus vehicle control groups (Fig. 3F). To determine the potential impact of more subtle transcriptional changes, GSEA was performed. Positive enrichment of several metabolism-related pathways, including carbohydrate, amino acid, and lipid metabolism, was noted following DHT exposure (Fig. 3G; Supplementary Table S4). However, the limited proliferative and transcriptional changes induced by DHT *in vitro* suggest that tumor-intrinsic AR signaling alone is insufficient to enhance male HNSCC growth *in vivo*.

### Sex-specific regulation of T-cell responses promotes sex biases in MOC1 tumor growth

**Figure 4.**
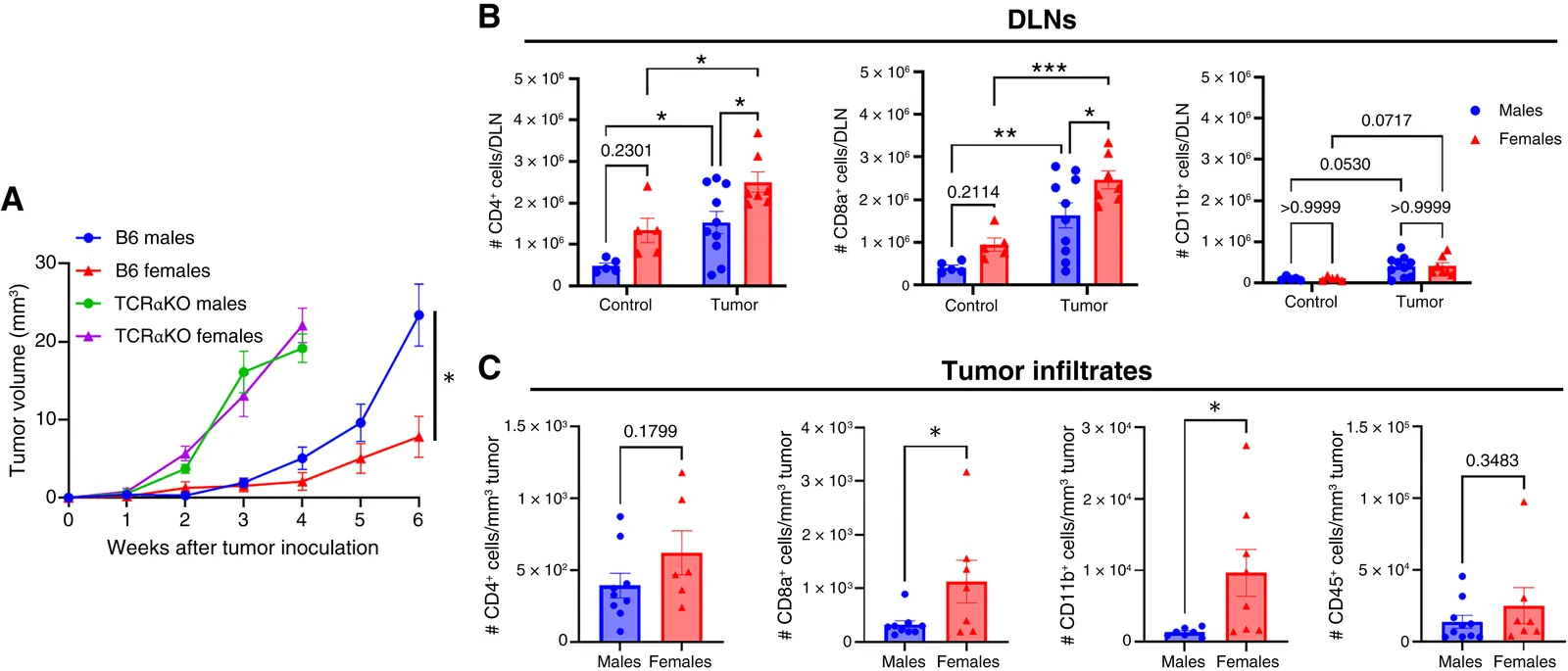
Sex-biased MOC1 tumor growth is T-cell dependent. **A,** MOC1 tumor volumes were assessed weekly in wild-type and TCRαKO mice. Immunophenotyping of the number of cells in (**B**) cervical DLNs and (**C**) MOC1 tumors of C57BL/6J mice was assessed by flow cytometry 6 weeks after tumor induction. **B** and **C,** Data are presented as the mean ± SEM with each data point representing an individual mouse and are representative of ≥2 independent experiments. **A,***n* ≥ 9 mice per group. Statistical analyses by the (**A** and **C**) unpaired *t* test or (**B**) two-way ANOVA followed by the Tukey multiple comparisons tests. *, *P* < 0.05; **, *P* < 0.01; ***, *P* < 0.001.

Given the limited impact of tumor-intrinsic androgen signaling on MOC1 cells and the critical role T cells play in controlling HNSCC onset and progression (37, 38), we next assessed whether sex differences in antitumor T-cell immunity promote HNSCC sex biases. MOC1 tumors were generated in TCRαKO mice, which lack both CD4^+^ and CD8^+^ T cells. As expected, C57BL/6J male mice developed larger tumors than female mice (Fig. 4A). However, this sex bias was eliminated in TCRαKO mice, implicating T cells in the sex-dependent tumor growth phenotype. To further characterize the role of T cells in HNSCC sex biases, MOC1-specific antitumor immunity was evaluated by flow cytometry (gating strategy provided in Supplementary Fig. S1). Although the relative frequencies of CD4^+^ T cells, CD8^+^ T cells, and CD11b^+^ myeloid cells were unaffected by tumor induction (Supplementary Fig. S2A), the absolute numbers of CD4^+^ and CD8^+^ T cells in the cervical DLNs of tumor-burdened mice were greater than those of non–tumor-bearing controls, regardless of sex, consistent with antitumor immune activation (Fig. 4B). A trend toward increased CD11b^+^ cell counts (males: *P* = 0.0530; females: *P* = 0.0717) was also noted (Fig. 4B). In comparison with MOC1 tumor-bearing females, tumor-bearing males possessed fewer DLN CD8^+^ T cells, as well as fewer (Fig. 4B) and reduced frequencies (Supplementary Fig. S2A) of CD4^+^ T cells in their tumor DLNs. CD11b^+^ myeloid cell numbers were similar in male and female tumor DLNs. Reduced CD8^+^ T-cell and CD11b^+^ myeloid cell infiltration was also noted in the MOC1 tumors of male versus female mice (Fig. 4C), despite the relative frequencies of these cells being similar between the sexes (Supplementary Fig. S2B). Sex did not influence the number or relative frequency of CD4^+^ T cells in MOC1 tumors in C57BL/6J mice (Fig. 4C; Supplementary Fig. S2B).

**Figure 5.**
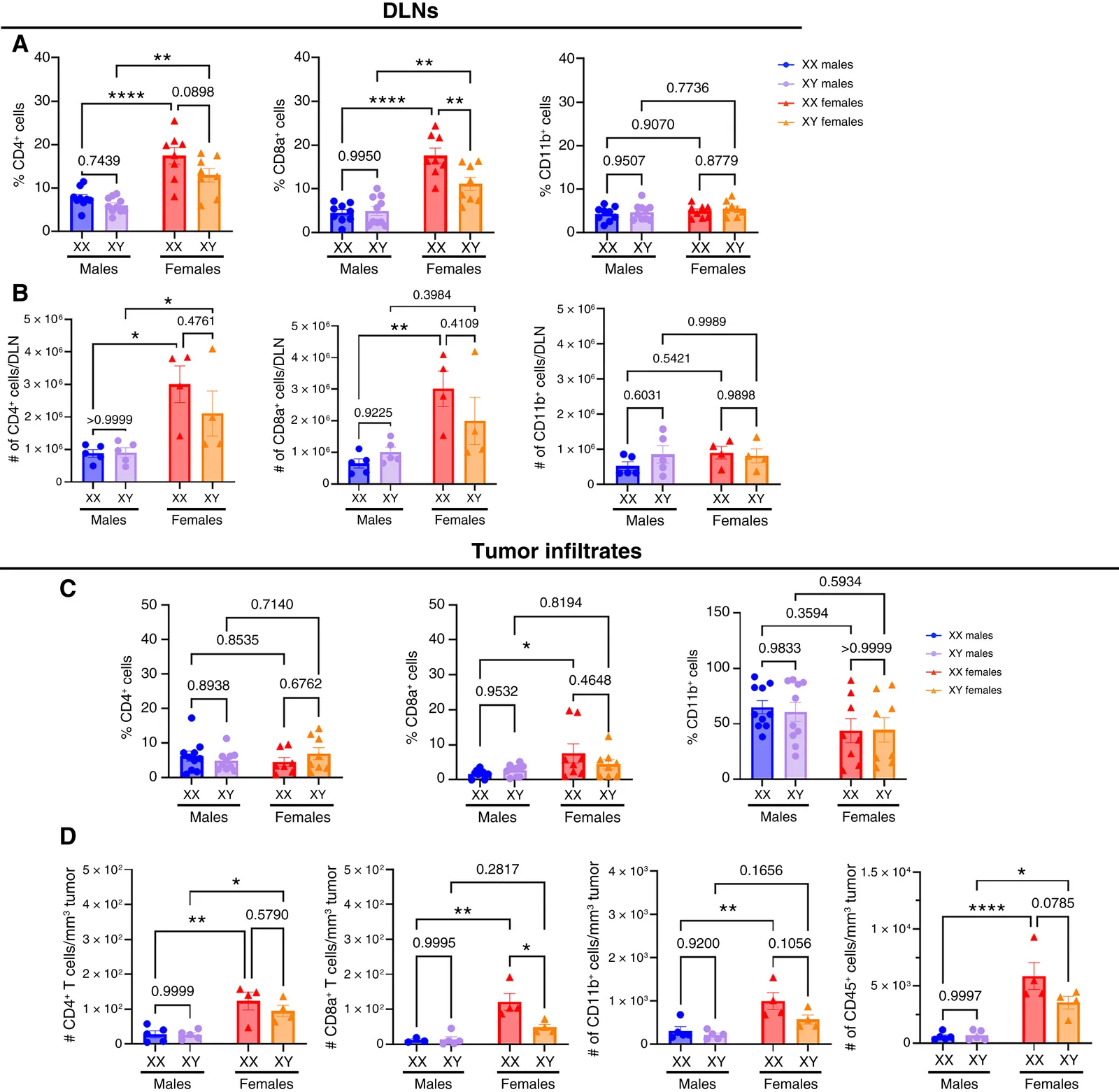
Male gonadal sex is associated with antitumor T-cell responses. Immunophenotyping of the (**A** and **C**) percent and (**B** and **D**) number of cells in (**A** and **B**) cervical DLNs and (**C** and **D**) MOC1 tumors of FCG mice was assessed by flow cytometry 5 weeks after tumor induction. Data are presented as the mean ± SEM with each data point representing an individual mouse and are representative of ≥2 independent experiments. Statistical analyses by two-way ANOVA followed by the Tukey multiple comparisons tests. *, *P* < 0.05; **, *P* < 0.01; ****, *P* < 0.0001.

The FCG model was then again utilized to evaluate the respective contributions of sex hormones and sex chromosomes to HNSCC phenotypes, this time to assess their influence on the relative frequency and, in a subset of animals, the absolute number of antitumor T cells. MOC1 tumor-bearing FCG gonadal males possessed a lower relative frequency of CD4^+^ and CD8^+^ T cells in their tumor DLNs compared with FCG gonadal females, as confirmed by two-way ANOVA (Fig. 5A; Supplementary Fig. S3A; Supplementary Table S5). Notably, a greater frequency of CD8^+^ T cells alongside a trend toward more CD4^+^ T cells (*P* = 0.0898) was observed in the DLNs of XXF versus XYF gonadal females (Fig. 5A). The absolute number of CD4^+^ T cells in the tumor DLNs was increased in XXF females versus XXM males and in XYF females versus XYM males (Fig. 5B). CD8^+^ T-cell counts in DLNs were also enhanced in XXF females versus XXM males but not in XYF females versus XYM males. Two-way ANOVA revealed an interactive effect of chromosomal and gonadal sex on CD8^+^ T-cell numbers (Supplementary Fig. S3B; Supplementary Table S5).

Although sex-specific factors did not modulate the relative frequencies of CD4^+^ T cells and CD8^+^ T cells in MOC1 tumors, CD11b^+^ myeloid cell frequencies were influenced by the main effect of gonadal sex (Fig. 5C; Supplementary Fig. S3C; Supplementary Table S5). The absolute number of CD45^+^ immune cell infiltrates into MOC1 tumors was found to be greater in gonadal males versus gonadal females of the same respective genotypes (Fig. 5D; Supplementary Fig. S3D; Supplementary Table S5). Two-way ANOVA confirmed the influence of gonadal sex on tumor-infiltrating CD4^+^ T-cell, CD8^+^ T-cell, and CD11b myeloid cell numbers (Supplementary Fig. S3D; Supplementary Table S5). Although sex chromosome complement did not statistically influence total CD45^+^ immune infiltration into the tumors of XXM versus XYM FCG males, XXF FCG females exhibited greater CD8^+^ T-cell densities than XYF FCG females (Fig. 5D). In addition, XXF FCG females consistently possessed greater numbers of total immune infiltrates (CD45^+^), CD4^+^ T cells, CD8^+^ T cells, and CD11b^+^ myeloid cells in comparison with XXM FCG males (Fig. 5D). Two-way ANOVA confirmed that XX-possessing animals had greater densities of CD8^+^ T cells and CD11b^+^ myeloid cells in their tumors in comparison with XY-possessing animals and also identified the interactive effect of gonadal sex and sex chromosome complement on the tumor-infiltrating CD8^+^ T-cell counts (Supplementary Fig. S3D; Supplementary Table S5). Similar results were observed in the 4-NQO model, in which 4-NQO–exposed FCG gonadal males induced weaker CD4^+^ T-cell, CD8^+^ T-cell, and CD11b^+^ myeloid cell responses in their cervical DLNs in comparison with FCG gonadal females (Supplementary Fig. S4; Supplementary Table S6). Taken together, these findings suggest that although male gonadal sex significantly limits antitumor T-cell responses, the collaborative influence of female gonadal sex and possession of an XX sex chromosome complement may support a more effective MOC1-specific T-cell response in XXF FCG females.

**Figure 6.**
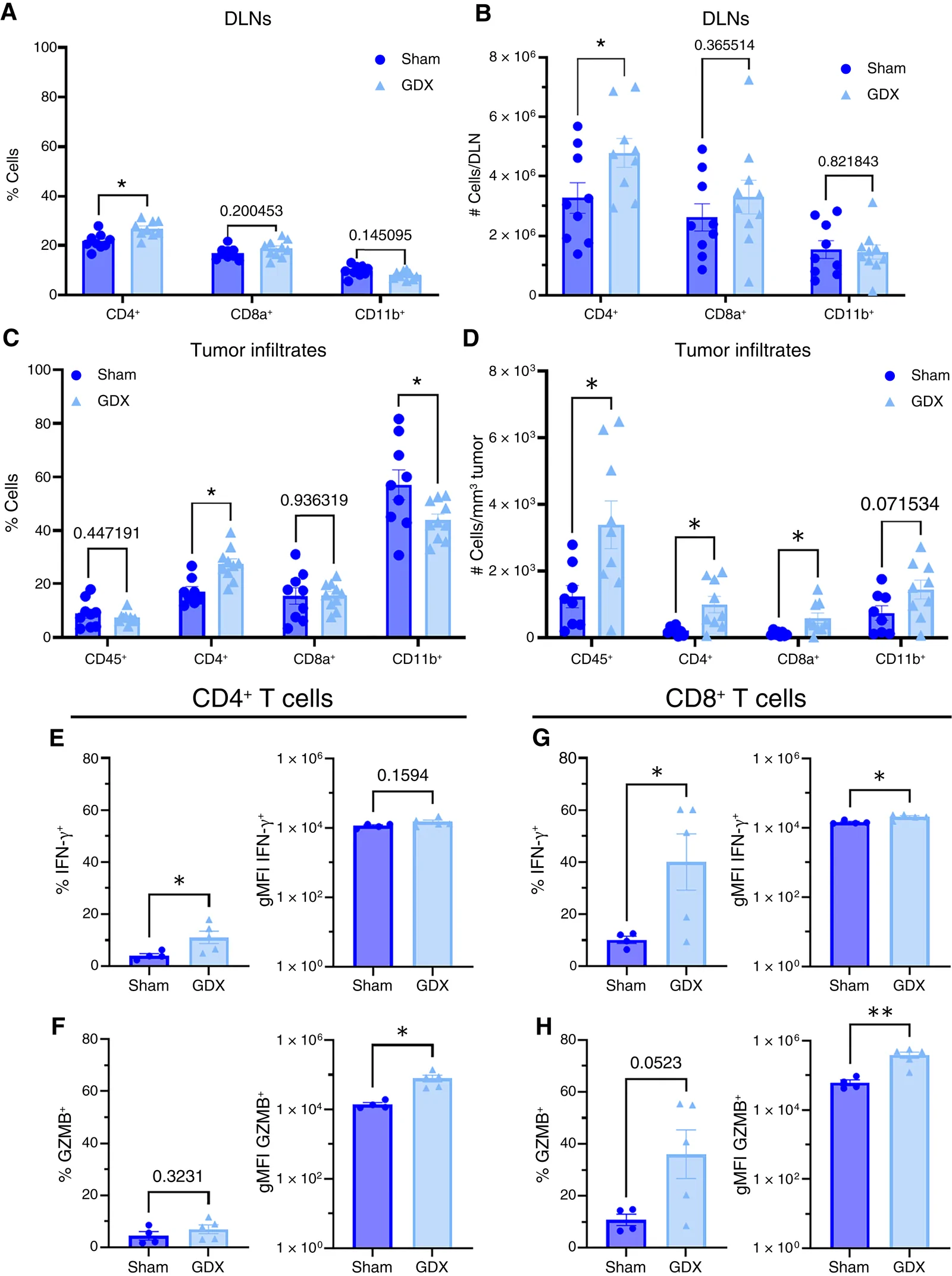
Castration enhances antitumor T-cell responses. Immunophenotyping of (**A** and **B**) tumor DLNs and (**C** and **D**) TILs isolated from sham-operated or castrated male C57BL/6J mice bearing MOC1 tumors 6 weeks after tumor induction. **E** and **F,** CD4^+^ T cells (CD45^+^CD4^+^) and (**G** and **H**) CD8^+^ T cells (CD45^+^CD8a^+^) infiltrating into MOC1 tumors were further analyzed to assess their activation status. The frequency (%) of CD4^+^ and CD8^+^ T cells expressing (**E** and **G**) IFN-γ and (**F** and **H**) granzyme B (GZMB) and the geometric mean fluorescence intensity (gMFI) were quantified for each marker. Data are represented as the mean ± SEM with each data point representing an individual mouse. Data are representative of ≥2 independent experiments. Statistical analyses by the unpaired *t* test. *, *P* < 0.05; **, *P* < 0.01.

As our *in vivo* studies demonstrated that androgens and male gonadal sex were associated with (i) enhanced MOC1 tumor growth (Figs. 1D and 2H) and (ii) reduced antitumor immune responses (Figs. 4 and 5), the causal influence of androgens in MOC1-specific T-cell function was then evaluated. The relative frequency and absolute number of MOC1-specific T cells in tumor DLNs and within MOC1 tumors were evaluated in sham-operated versus castrated C57BL/6J male mice. Castration increased the relative frequency and number of CD4^+^ T cells in the DLNs of MOC1-bearing mice but had no effect on DLN CD8^+^ T-cell or CD11b^+^ myeloid cell populations (Fig. 6A and B). Castration also increased the frequency of CD4^+^ T cells in MOC1 tumors (Fig. 6C) and significantly enhanced the number of tumor-infiltrating CD45^+^ leukocytes (Fig. 6D). Significant increases in CD4^+^ and CD8^+^ T-cell densities, as well as a trend toward increased CD11b^+^ myeloid cell counts (*P* = 0.071534), were noted in the tumors of castrated versus sham-operated males. Ovariectomy of females did not alter DLN or TIL populations (Supplementary Fig. S5). Tumor-infiltrating T cells from castrated males also exhibited a more active phenotype, demonstrated by a higher frequency of IFN-γ–expressing cells (Fig. 6E and G) and increased granzyme B expression (Fig. 6F and H). Together, these data confirm recent studies (39) suggesting that in males, androgens promote HNSCC tumor growth by suppressing antitumor T-cell infiltration and activity.

## Discussion

Sex differences in HNSCC incidence and mortality are well established (40). Male-biased sex differences in HNSCC incidence and survival have previously been attributed to sex-specific lifestyle risks like tobacco and alcohol exposure. However, even after adjustment for these risk factors, males exhibit worse outcomes than females (41), suggesting that sex-specific biological factors influence HNSCC onset and progression. Understanding the biological mechanisms underlying these biases is crucial for the development of novel therapies and improvement of current treatment strategies. Both clinical and preclinical reports have previously implicated sex hormone receptor expression and signaling in HNSCC sex biases (42, 43). For example, females undergoing postmenopausal hormone replacement therapy are at an almost twofold reduced risk for developing HNSCC (44). In contrast, a clinical benefit rate of nearly 75% was reported in male patients with salivary gland carcinoma who received androgen deprivation therapy (ADT; ref. 45). Evidence of sex chromosome–dependent effects on HNSCC prognosis is not as well understood; however, loss of Y chromosome complement in male patients with HNSCC is associated with decreased survival (46). As such, sex hormones and sex chromosome complement have been proposed as mechanistic drivers of HNSCC via tumor-intrinsic mechanisms. Expanding upon these reports, here we evaluated the individual and collaborative influence of sex chromosome complement and sex hormones on cancer risk via the modulation of tumor-extrinsic pathways, including TIME regulation. Consistent with human epidemiologic data (47) and previous reports of higher oral and esophageal tumor incidence in 4-NQO–exposed males (48, 49), we observed greater susceptibility and accelerated disease progression in males following 4-NQO exposure and in the MOC1 syngeneic tumor model. Interestingly, although the combined influence of gonadal sex and possession of an XX sex chromosome complement was associated with enhanced tumor T-cell responses in females, androgen-mediated T-cell suppression was identified as a key driver of cancer onset and progression in males.

Robust antitumor immune responses are critical for limiting HNSCC onset and progression. Clinically, high intratumoral densities of CD8^+^ T cells and NK cells are associated with improved outcomes, whereas immunosuppressive TIMEs correlate with increased incidence and poorer prognosis (37, 38). Recent bulk RNA and single-cell transcriptomic analyses have identified sex-specific transcriptional shifts in the TIME of human patients with HNSCC, with immunosuppressive transcriptional signatures being enriched in male tumors (39, 50). Consistent with these reports and data recently published by Wang and colleagues (39), we demonstrate impaired T-cell responses in the cervical DLNs of MOC1 tumor-bearing and 4-NQO–exposed males, as well as reduced T-cell infiltration into male versus female MOC1 tumors. Antitumor immunity was found to drive sex biases in MOC1 tumor growth, both here and in the Wang and colleagues (39) study, as sex differences in MOC1 tumor growth were eliminated in mice lacking both CD4^+^ and CD8^+^ T cells. Jagadeesha and colleagues (51) recently generated similar results in an orthotopic MOC2 syngeneic model. Although Horan and colleagues (52) previously reported sex-biased immunologic phenotypes in MOC1 syngeneic tumors, the magnitudes of their reported immunologic sex differences were smaller than those reported here, and no sex difference in tumor growth was detected in their study. This may be the result of differences in the tumor induction protocols used between studies. Whereas the use of oral orthotopic (current report) versus subcutaneous flank tumors (39) had no impact on sex-biased tumor growth, the use of Matrigel in the Horan and colleagues study may have. Notably, the use of Matrigel has previously been demonstrated to significantly reduce the percentage of tumor-infiltrating IFN-γ^+^ CD4^+^ and CD8^+^ T cells in other preclinical cancer models (53). Given the T-cell dependency of MOC1 sex-biased growth, reduced T-cell infiltration into Matrigel-containing MOC1 tumors may explain the lack of sex differences noted in the Horan and colleagues report, which utilized Matrigel during orthotopic tumor implantation, and further support the important role T cells play in HNSCC sex biases.

AR is expressed by multiple immune cell populations, including T and B lymphocytes, macrophages, and neutrophils (54). AR signaling has been shown to suppress effector T-cell responses and enhance Treg function in a variety of diseases (13, 14, 55). Given recent reports which illustrate the role of androgens in restraining antitumor T-cell function (12–14, 39, 56), we hypothesized that androgen-dependent regulation of antitumor immunity would promote tumor growth in males. Consistent with data demonstrating that ADT enhances effector T-cell responses in prostate cancer by blocking AR-mediated transcriptional repression of *Ifng* (13) and recent findings that ADT efficacy in HNSCC preclinical models was CD8^+^ T-cell dependent (39), castration not only reduced MOC1 tumor size but also enhanced antitumor T-cell responses. Here, DLN CD4^+^ T-cell frequencies and the number and activation state of MOC1 tumor-infiltrating CD4^+^ and CD8^+^ T cells were increased in castrated versus sham-operated males. Recent studies by Wang and colleagues (39) confirm that this androgen deprivation–mediated effect is not model specific, as similar results were observed in response to 4-NQO exposure. Interestingly, although androgen signaling has been reported to repress MHC I expression, resulting in reduced CD8^+^ T-cell activation (57), no changes in H-2Kb or PD-L1 expression were identified by RNA-seq or flow cytometric staining (Supplementary Fig. S6) of DHT-treated MOC1 cells *in vitro*. Future studies utilizing T cell–specific AR knockouts will provide valuable insights into the possible causative link between T cell–intrinsic AR signaling and androgen-mediated T-cell suppression in HNSCC. Additional indirect immunomodulatory mechanisms that should be explored include androgen-mediated shifts in tumor microenvironment (TME) myeloid cell phenotypes (58) and altered immunomodulatory gut microbiome populations in males (21).

AR is reported to be expressed in about 70% of laryngeal and salivary duct tumors and is expressed in several human HNSCC cell lines (42). Although androgens promote proliferation and metabolic reprogramming in HNSCC cell lines and other tumor cells (3, 59), here we report that DHT exposure did not increase *in vitro* proliferation of MOC1 cells and induced only subtle gene-level transcriptional changes. However, GSEA identified enrichment in metabolic pathways, including glycolysis, pentose phosphate pathways, amino acid and lipid metabolism pathways, and mTOR signaling. This pattern suggests a coordinated shift across many genes within shared metabolic networks that are not apparent at the individual gene level. These findings raise the possibility that AR signaling supports MOC1 tumor growth through global shifts in glycolytic reprogramming and biosynthetic precursor anabolism, rather than direct proliferative effects. This interpretation is consistent with prior reports that AR signaling upregulates glycolysis machinery, increases glucose uptake, and activates mTOR signaling to promote tumor growth (60). The absence of a proliferative response to DHT *in vitro* may reflect the limitation of this system to recapitulate the *in vivo* effects of androgens on tumor growth. Alternately, the inconsistency between metabolic pathway enrichment and proliferation may be specific to MOC1 cells. Future studies knocking out AR in MOC1 cells will clarify the direct effect of AR signaling on MOC1 tumor growth *in vivo*.

The current study expands on previous reports investigating sex hormone–mediated influences in antitumor immunity by also evaluating whether sex chromosome complement contributes to sex-biased HNSCC tumorigenesis and antitumor immunity using the FCG mouse model. The biallelic expression of XLIR genes has previously been demonstrated to enhance immune activation in an XX-dependent manner (21, 61, 62). Although we did not observe enhanced antitumor responses in MOC1 tumor-bearing XXM versus XYM FCG males, MOC1 tumor-bearing XXF FCG females exhibited greater relative frequencies of CD8^+^ T cells in their tumor DLNs and more tumor-infiltrating CD8^+^ T cells compared with XYF FCG females. These sex-specific differences in sex chromosome dependency likely reflect the collaborative impact of estrogen signaling and XX sex chromosome complement in female immune cells. This raises the possibility that, in females, ER signaling mediates enhanced XLIR expression in XX cells through its binding to estrogen response elements in XLIR gene promoters (8). Notably, we did not observe any impacts of ovariectomy on female antitumor immune responses. As estrogen seemingly influenced tumor-specific T-cell responses in XXF FCG females, but not in C57BL/6J females, it is possible that ER signaling is disparately regulated between the two models. It is generally accepted that XX- and XY-possessing mice of the same gonadal sex possess similar circulating hormone levels (23), and ERα and ERβ expression differs in mice with ovaries versus testes. However, we are unaware of any previous report assessing ERα and ERβ expression levels in XXF versus XYF female FCG mice. Differential ER expression could explain differences in estrogen responsiveness between our models. Ovariectomy-induced shifts in circulating androgen levels (63) could also contribute; however, ovariectomy (OVX) did not affect DLN T-cell numbers or tumor infiltration in our studies. Given that we did not assess T-cell activity or innate immune subpopulations following OVX, it is possible that estrogens influence antitumor immunity in a manner outside of what we observed. Thus, additional studies are needed to define the relative contributions of X-linked gene dosage, estrogen-dependent regulation of XLIRs, and ER signaling in HNSCC T-cell immunity. Importantly, when interpreting sex chromosome–dependent influences using the FCG model, the impact of recently identified X-linked gene translocations should be taken into consideration, as this translocation events affect the strain used in this study. Nine X-linked genes, including the XLIR genes, *Tlr7* and *Tlr8* (64) that have previously been implicated in enhanced antitumor immunity (65), reportedly translocated to the Y^*Sry*−^ chromosome. In the current study, XLIR gene translocation could attenuate XX- versus XY-dependent immune phenotypes, due to the overexpression of XLIR in XY-possessing immune cells.

Sex differences in antitumor immunity may also influence HNSCC immunotherapeutic efficacy. Immune checkpoint inhibitors (ICI) are approved for the treatment of recurrent and metastatic disease (66). Evidence suggests that males derive greater benefit from their usage. This may reflect a more immunosuppressive TME in males that is more responsive to ICIs and/or enhanced immunoediting in females, leading to less immunogenic tumors. Multiple reports have now demonstrated that T cell–intrinsic AR signaling limits CD8^+^ T-cell cytotoxicity and that ADT synergized with ICIs to improve outcomes in HNSCC and other preclinical cancer models (13, 39). Consistent with these findings, our data support the potential utility of combination ADT/ICI use in HNSCC and highlights the need to consider sex-specific immune differences in treatment design. Given that females also produce androgens, albeit at lower levels (67), ADT may also enhance female antitumor responses. In addition, although the current report focuses on human papillomavirus–negative HNSCC sex biases, these data may also extend to human papillomavirus–positive HNSCC, which exhibits similar sex biases and is known to induce strong antitumor responses (68).

Taken together, our results identify androgens as a key mediator of HNSCC sex biases, functioning to suppress antitumor T-cell responses. This study highlights the importance of understanding the basic contribution sex-specific biological factors have in HNSCC onset and progression and identifies androgen signaling as a potential therapeutic target to enhance tumor-specific immunity in patients with HNSCC.

## Supplementary Material

Table S1 Table S1. Primers utilized for FCG mouse genotyping (Invitrogen).

Table S2 Table S2. Effects of gonadal sex on 4-NQO carcinogenesis in Figure 1A-C.

Table S3 Table S3. Main effects of sex-specific factors in Figure 2.

Table S4 Table S4. Pathways enriched in MOC1 cells following in vitro DHT exposure.

Table S5 Table S5. Main effects of sex-specific factors in Figure 5.

Table S6 Table S6. Main effects of sex-specific factors in Figure S4.

Figure S1 Figure S1. Flow cytometry gating schema for phenotypic analysis experiments.

Figure S2 Figure S2. MOC1-bearing males demonstrate weaker anti-tumor immune responses.

Figure S3 Figure S3. Main effects of sex chromosome complement and gonadal sex on anti-tumor immunity in MOC1 tumor-bearing FCG mice.

Figure S4 Figure S4. FCG gonadal males exhibit weaker immune cell activation in response to 4-NQO exposure.

Figure S5 Figure S5. Ovariectomy does not impact female anti-tumor immunity.

Figure S6 Figure S6. Androgens do not impact H-2Kb or PD-L1 expression in MOC1 cells

## Data Availability

The RNA-seq data have been deposited to Gene Expression Omnibus (accession #GSE320472). All other datasets are available from the corresponding author upon reasonable request.
