# Supplementary material for: Androgen-Mediated T-cell Suppression Drives Sex Bias in Head and Neck Squamous Cell Carcinoma
Source: Cancer Res Commun. 2026 Nov 3;6(11):2578–92. doi: 10.1158/2767-9764.CRC-26-0244 (PMC13729707; doi:10.1158/2767-9764.CRC-26-0244)
Supplement: Supplementary file 7 [file crc-26-0244_table_s1_suppst1.docx]

Table S1. Primers utilized for FCG mouse genotyping (Invitrogen).

| **Target** | **Sequence** |
| --- | --- |
| *Myo* | Forward: 5ʹ-TTA CGT CCA TCG TGG ACA GCA T-3ʹ  Reverse: 3ʹ-TGG GCT GGG TGT TAG TCT TAT-5ʹ |
| *Sry* | Forward: 5ʹ-AGC CCT ACA GCC ACA TGA TA-3ʹ  Reserve: 3ʹ- TTG CCT GTA TGT GAT GG-5ʹ |
| *Ymt* | Forward: 5ʹ- GAG CTC TAC AGT GAT GA-3ʹ  Reverse: 3ʹ-CAG TTA CCA ATC AAC ACA TCA C-5ʹ |
