# Supplementary material for: Androgen-Mediated T-cell Suppression Drives Sex Bias in Head and Neck Squamous Cell Carcinoma
Source: Cancer Res Commun. 2026 Nov 3;6(11):2578–92. doi: 10.1158/2767-9764.CRC-26-0244 (PMC13729707; doi:10.1158/2767-9764.CRC-26-0244)
Supplement: Supplementary file 8 [file crc-26-0244_table_s2_suppst2.docx]

Table S2. Effects of gonadal sex on 4-NQO carcinogenesis in Figure 1A-C.

| ***Sex*** | ***% Lesion-Free at Endpoint*** | ***Average # of Lesions >0.5 mm*** | ***Average Lesion Area (mm^2^)*** |
| --- | --- | --- | --- |
| *Male* | 0/10 | 1.5 ± 0.71 | 5.63889 ± 3.92132 mm^2^ |
| *Female* | 4/10 | 0.6 ± 0.52 | 2.36111 ± 1.17998 mm^2^ |

*The influence of gonadal sex on (Fig. 1A) percent of mice lesion-free at endpoint, (Fig. 1B) average number ± standard deviation of lesions per mouse >0.5 mm in diameter at 22-week timepoint, and (Fig. 1C) average lesion area ± standard deviation at 22-week endpoint of 4-NQO-exposed mice as visualized in Fig. 1A-C.*
