# Supplementary material for: Androgen-Mediated T-cell Suppression Drives Sex Bias in Head and Neck Squamous Cell Carcinoma
Source: Cancer Res Commun. 2026 Nov 3;6(11):2578–92. doi: 10.1158/2767-9764.CRC-26-0244 (PMC13729707; doi:10.1158/2767-9764.CRC-26-0244)
Supplement: Supplementary file 9 [file crc-26-0244_table_s3_suppst3.docx]

Table S3. Main effects of sex-specific factors in Figure 2.

| ***Fig. 2*** | ***Fig. 2C***  ***Week of Lesion Onset*** | | ***Fig. 2E***  ***Lesion Area (mm^2^)*** | | ***Fig. 2H***  ***Tumor Volume (mm^3^)*** | |
| --- | --- | --- | --- | --- | --- | --- |
| ***Effect*** | ***F*** | ***p value*** | ***F*** | ***p value*** | ***F*** | ***p value*** |
| *Main Effects* |  |  |  |  |  |  |
| Gonadal Sex (F, M) | **37.34** | **<0.0001** | **17.20** | **0.0003** | **8.813** | **0.0056** |
| Chromosomes (XX, XY) | 1.077 | 0.3063 | 2.648 | 0.1149 | 0.3507 | 0.5579 |
| *Two-way Interaction* |  |  |  |  |  |  |
| Gonadal*Chromosomes | 1.609 | 0.2128 | 0.0755 | 0.7856 | **5.202** | **0.0294** |

Summary of the two-way ANOVA results investigating gonadal sex and chromosomal main effects on (Fig. 2C, F) week of lesion onset and (Fig. 2E, G) lesion area readouts in 4-NQO-exposed mice, and (Fig. 2H, I) tumor volume measurements in MOC1 tumor-bearing mice. Two-way interactions are also included for each readout. Bold values indicate significance (p<0.05).
