# Supplementary material for: Androgen-Mediated T-cell Suppression Drives Sex Bias in Head and Neck Squamous Cell Carcinoma
Source: Cancer Res Commun. 2026 Nov 3;6(11):2578–92. doi: 10.1158/2767-9764.CRC-26-0244 (PMC13729707; doi:10.1158/2767-9764.CRC-26-0244)
Supplement: Supplementary file 10 [file crc-26-0244_table_s4_suppst4.docx]

**Table S4. Pathways enriched in MOC1 cells following *in vitro* DHT exposure.**

| **Rank** | **KEGG term** | **NES** | **FDR q-val** |
| --- | --- | --- | --- |
| 1 | KEGG_GLYCOLYSIS_GLUCONEOGENESIS | 2.09 | 0.004 |
| 2 | KEGG_PENTOSE_PHOSPHATE_PATHWAY | 1.99 | 0.011 |
| 3 | KEGG_FRUCTOSE_AND_MANNOSE_METABOLISM | 1.96 | 0.013 |
| 4 | KEGG_PYRUVATE_METABOLISM | 1.95 | 0.01 |
| 5 | KEGG_BUTANOATE_METABOLISM | 1.83 | 0.03 |
| 6 | KEGG_VALINE_LEUCINE_AND_ISOLEUCINE_DEGRADATION | 1.83 | 0.027 |
| 7 | KEGG_CIRCADIAN_RHYTHM_MAMMAL | 1.83 | 0.025 |
| 8 | KEGG_MTOR_SIGNALING_PATHWAY | 1.81 | 0.028 |
| 9 | KEGG_FATTY_ACID_METABOLISM | 1.8 | 0.029 |
| 10 | KEGG_STARCH_AND_SUCROSE_METABOLISM | 1.8 | 0.027 |
| 11 | KEGG_ARGININE_AND_PROLINE_METABOLISM | 1.78 | 0.032 |
| 12 | KEGG_PROPANOATE_METABOLISM | 1.78 | 0.03 |
| 13 | KEGG_GALACTOSE_METABOLISM | 1.77 | 0.031 |
| 14 | KEGG_GLUTATHIONE_METABOLISM | 1.76 | 0.031 |

*Summary of normalized enrichment scores and false discovery rate q-values of pathways enriched in MOC1 cells following exposure to DHT as visualized in Figure 3G. Gene set enrichment analysis was done with GSEA (v4.2.3) based on gene expression changes against DEGG gene set from MSigDB (v2023). Only expressed genes (RPKM>3 at least in one compared condition) were included.*
