# Supplementary material for: Androgen-Mediated T-cell Suppression Drives Sex Bias in Head and Neck Squamous Cell Carcinoma
Source: Cancer Res Commun. 2026 Nov 3;6(11):2578–92. doi: 10.1158/2767-9764.CRC-26-0244 (PMC13729707; doi:10.1158/2767-9764.CRC-26-0244)
Supplement: Supplementary file 11 [file crc-26-0244_table_s5_suppst5.docx]

Table S5. Main effects of sex-specific factors in Figure 5.

| ***Fig. 5A*** | ***% CD4+ Cells*** | | | | ***% CD8a+ Cells*** | | | | ***% CD11b+ Cells*** | | | |
| --- | --- | --- | --- | --- | --- | --- | --- | --- | --- | --- | --- | --- |
| ***Effect*** | ***F*** | | ***p value*** | | ***F*** | | ***p value*** | | ***F*** | | ***p value*** | |
| *Main Effects* |  | |  | |  | |  | |  | |  | |
| Gonadal Sex (F, M) | **44.47** | | **<0.0001** | | **59.30** | | **<0.0001** | | 1.323 | | 0.2589 | |
| Chromosomes (XX, XY) | **6.167** | | **0.0186** | | **5.821** | | **0.0219** | | 0.8254 | | 0.3706 | |
| *Two-way Interaction* |  | |  | |  | |  | |  | |  | |
| Gonadal*Chromosomes | 1.242 | | 0.2737 | | **7.499** | | **0.0101** | | 0.03575 | | 0.6513 | |
|  | | | | | | | | | | | | |
| ***Fig.5B*** | ***# CD4+ Cells/DLN*** | | | | ***# CD8a+ Cells/DLN*** | | | | ***# CD11b+ Cells/DLN*** | | | |
| ***Effect*** | ***F*** | | ***p value*** | | ***F*** | | ***p value*** | | ***F*** | | ***p value*** | |
| *Main Effects* |  | |  | |  | |  | |  | |  | |
| Gonadal Sex (F, M) | **16.72** | | **0.0011** | | **15.22** | | **0.0016** | | 0.7393 | | 0.4044 | |
| Chromosomes (XX, XY) | 1.136 | | 0.3045 | | 0.6002 | | 0.4514 | | 0.3736 | | 0.5509 | |
| *Two-way Interaction* |  | |  | |  | |  | |  | |  | |
| Gonadal*Chromosomes | 1.290 | | 0.2752 | | 2.584 | | 0.1303 | | 1.134 | | 0.3050 | |
|  | | | | | | | | | | | | |
| ***Fig. 5C*** | ***% CD4+ Cells*** | | | | ***% CD8a+ Cells*** | | | | ***% CD11b+ Cells*** | | | |
| ***Effect*** | ***F*** | | ***p value*** | | ***F*** | | ***p value*** | | ***F*** | | ***p value*** | |
| *Main Effects* |  | |  | |  | |  | |  | |  | |
| Gonadal Sex (F, M) | 0.02574 | | 0.8736 | | **7.055** | | **0.0124** | | **4.276** | | **0.0468** | |
| Chromosomes (XX, XY) | 0.1526 | | 0.6987 | | 0.5361 | | 0.4695 | | 0.04014 | | 0.8425 | |
| *Two-way Interaction* |  | |  | |  | |  | |  | |  | |
| Gonadal*Chromosomes | 0.1727 | | 0.1984 | | 2.069 | | 0.1603 | | 0.08052 | | 0.7784 | |
|  | | | | | | | | | | | | |
| ***Fig. 5D*** | ***# CD45+ Cells/mm^3^ tumor*** | | | ***# CD4+ Cells/mm^3^ tumor*** | | | ***# CD8a+ Cells/mm^3^ tumor*** | | | ***# CD11b+ Cells/mm^3^ tumor*** | | |
| ***Effect*** | ***F*** | ***p value*** | | ***F*** | | ***p value*** | ***F*** | ***p value*** | | ***F*** | | ***p value*** |
| *Main Effects* |  |  | |  | |  |  |  | |  | |  |
| Gonadal Sex (F, M) | **48.58** | **<0.0001** | | **30.68** | | **<0.0001** | **26.91** | **0.0002** | | **20.91** | | **0.0004** |
| Chromosomes (XX, XY) | 3.668 | 0.0761 | | 1.032 | | 0.3269 | **6.427** | **0.0262** | | **5.180** | | **0.0391** |
| *Two-way Interaction* |  |  | |  | |  |  |  | |  | |  |
| Gonadal*Chromosomes | 4.183 | 0.0601 | | 0.8444 | | 0.3737 | **7.238** | **0.0197** | | 2.054 | | 0.1738 |

*Summary of the two-way ANOVA results investigating gonadal sex and chromosomal main effects on (Fig. 5A; Fig. S2A) percent and (Fig. 5B; Fig. S2B) number of cells per DLN and (Fig. 5C; Fig. S2C) percent and (Fig. 5D; Fig. S2D) density of tumor-infiltrating leukocytes in MOC1 tumor-bearing mice. Two-way interactions are also included for each readout. Bold values indicate significance (p<0.05).*
