# Supplementary material for: Androgen-Mediated T-cell Suppression Drives Sex Bias in Head and Neck Squamous Cell Carcinoma
Source: Cancer Res Commun. 2026 Nov 3;6(11):2578–92. doi: 10.1158/2767-9764.CRC-26-0244 (PMC13729707; doi:10.1158/2767-9764.CRC-26-0244)
Supplement: Supplementary file 12 [file crc-26-0244_table_s6_suppst6.docx]

Table S6. Main effects of sex-specific factors in Figure S4.

| ***Fig. S4A*** | ***% CD4+ Cells*** | | ***% CD8a+ Cells*** | | ***% CD11b+ Cells*** | |
| --- | --- | --- | --- | --- | --- | --- |
| ***Effect*** | ***F*** | ***p value*** | ***F*** | ***p value*** | ***F*** | ***p value*** |
| *Main Effects* |  |  |  |  |  |  |
| Gonadal Sex (F, M) | **7.581** | **0.0098** | **26.14** | **<0.0001** | **56.20** | **<0.0001** |
| Chromosomes (XX, XY) | **12.37** | **0.0013** | 2.466 | 0.1259 | 1.393 | 0.2463 |
| *Two-way Interaction* |  |  |  |  |  |  |
| Gonadal*Chromosomes | 1.748 | 0.1953 | 2.040 | 0.1626 | 2.431 | 0.1285 |
| ***Fig. S4C*** | ***# CD4+ Cells/DLN*** | | ***# CD8a+ Cells/DLN*** | | ***# CD11b+ Cells/DLN*** | |
| ***Effect*** | ***F*** | ***p value*** | ***F*** | ***p value*** | ***F*** | ***p value*** |
| *Main Effects* |  |  |  |  |  |  |
| Gonadal Sex (F, M) | **6.462** | **0.0159** | **23.22** | **<0.0001** | **28.59** | **<0.0001** |
| Chromosomes (XX, XY) | 0.01966 | 0.8893 | 0.1299 | 0.7208 | **7.544** | **0.0097** |
| *Two-way Interaction* |  |  |  |  |  |  |
| Gonadal*Chromosomes | 0.4496 | 0.5072 | 0.003619 | 0.9524 | **4.676** | **0.0379** |

*Summary of the two-way ANOVA results investigating gonadal sex and chromosomal main effects on (Fig. S4A, B) percent and (Fig. S4C, D) number of cells per DLN in 4-NQO-exposed mice. Two-way interactions are also included for each readout. Bold values indicate significance (p<0.05).*
